# Carboxymethyl benzylamide dextran inhibits angiogenesis and growth of VEGF-overexpressing human epidermoid carcinoma xenograft in nude mice

**DOI:** 10.1038/sj.bjc.6601029

**Published:** 2003-07-01

**Authors:** Y Hamma-Kourbali, A Starzec, R Vassy, A Martin, M Kraemer, G Perret, M Crépin

**Affiliations:** 1 Laboratoire de Ciblage Fonctionnel des Tumeurs Solides, UPRES 2360, Faculté de Médecine, Université Paris 13, 74 rue Marcel Cachin, 93017 Bobigny cedex, France; 2 Service d'Anatomie Pathologie, Hôpital Avicenne, 125, route de Stalingrad, F-93017 Bobigny cedex, France; 3 Laboratoire d'Hémostase, Endothélium et Angiogénèse, Unité INSERM 553, Hôpital Saint-Louis, 75010 Paris, France

**Keywords:** dextran derivative, vascular endothelial growth factor (VEGF), epidermoid carcinoma A431 cells, angiogenesis

## Abstract

Vascular endothelial growth factor (VEGF) expression is elevated in a wide variety of solid tumours. Inhibition of VEGF activities is able to reduce angiogenesis and tumour growth. We have recently shown *in vitro* that carboxymethyl dextran benzylamide (CMDB7) prevents the binding of VEGF_165_ to its cell surface receptors and thus inhibits VEGF activities on endothelial cells. In the present study, we explored the effects of CMDB7 on highly aggressive human epidermoid carcinoma A431 cells known to overexpress epidermal growth factor receptors (EGFRs) and produce a high amount of VEGF and a minor quantity of bFGF. *In vitro,* CMDB7 blocked the mitogenic activity of A431-conditioned medium on endothelial cells. Concerning A431 cells, CMDB7 inhibited their proliferation and the VEGF_165_ binding to them. *In vivo*, administration of CMDB7 (10 mg kg^−1^) three times per week for 2 weeks inhibited the growth of A431 xenografts in nude mice by 73% as compared to the control group. Immunostaining of endothelial cells with mouse-specific GSL-1 lectin in tumour sections revealed that CMDB7 also inhibited the density of intratumour endothelial cells by 66%. These findings demonstrate that CMDB7 has an efficient antiangiogenic and antitumour action *in vivo* even when tumour cells produce a high level of VEGF and EGFRs.

Neovascularisation is critical for supporting the rapid growth of solid tumours (Folkman, 1990). Tumour angiogenesis appears to be achieved by the overexpression of angiogenic agents within solid tumours that stimulate host vascular endothelial cell mitogenesis and possibly chemotaxis. It is well established now that the induction of vascular endothelial growth factor (VEGF) expression, including via tumour hypoxia (Hlatky *et al*, 1994), plays a major role in tumour angiogenesis (Dvorak *et al*, 1991; Millauer *et al*, 1994; Goldman *et al*, 1998). In recent years, it has been widely shown that VEGF activity is a key feature during tumour growth and angiogenesis, and that blocking of this signal transduction pathway may inhibit tumour progression (Cheng *et al*, 1996; Relf *et al*, 1997). *In vivo*, VEGFs act as potent mitogenic factors for endothelial cells and as blood vessel permeabilising agents (Senger *et al*, 1983; Plouët *et al*, 1989; Klagsbrun and Soker, 1993; Yuan *et al*, 1996). The VEGF gene family currently includes six members: VEGF-A (prototype VEGF), placenta growth factor (PlGF), VEGF-B, VEGF-C, VEGF-D and VEGF-E, (produced by Orf virus), (reviewed by Veikkola *et al*, 2000). VEGF is a homodimeric glycoprotein that exists in six isoforms containing 121, 145, 162, 165, 189 and 206 amino-acid residues as a result of alternative splicing from a single gene (Ferrara and Davis-Smith, 1997; Lange *et al*, 2003). The predominant and the best characterised VEGF species is the heparin-binding 165-amino acid-long form VEGF_165_ (reviewed by Neufeld *et al*, 1999).

Three receptors with tyrosine kinase activities have been identified as VEGF receptors: VEGFR-1 (Flt-1) (deVries *et al*, 1992), VEGFR-2 (KDR/Flk-1) (Terman *et al*, 1992), proteins with apparent molecular weights of 180 and 230 kDa, respectively, and VEGFR-3 (Kaipainen *et al*, 1995). They have been shown to bind VEGF with high affinity. Recently, an additional binding site, neuropilin-1 (NP-1), was identified and shown to be expressed on the surface of endothelial and tumour cells (Soker *et al*, 1998). NP-1 modulates the binding of VEGF_165_ to VEGFR-2 acting as a coreceptor that enhances VEGF_165_-induced activities mediated by VEGFR-2 (Whitaker *et al*, 2001; Soker *et al*, 2002).

Carboxymethyl dextran benzylamide (CMDB7) is a noncytotoxic substituted dextran. We have recently shown *in vitro* that it prevents the binding of VEGF_165_ to human umbilical vein endothelial cell surface and thus inhibits VEGF_165_-induced phosphorylation of VEGFR-2 and consequently endothelial cell proliferation (Hamma-Kourbali *et al*, 2001). In the present study, we explored *in vitro* and *in vivo* the effects of CMDB7 on human epidermoid carcinoma A431 cells known to produce a high amount of VEGF and a minor quantity of bFGF (Myoken *et al*, 1991). The other peculiarity of A431 cells is the production of a newly identified splice form of VEGF, VEGF-162, which binds more efficiently than VEGF-165 to a natural basement membrane of endothelial cells (Lange *et al*, 2003). Moreover, A431 cells express a high level of epidermal growth factor receptors (EGFRs) activated by EGF, a nonheparin-binding growth factor, which does not interact with CMDB7 (Bagheri-Yarmand *et al*, 1998b). Interestingly, the resistance of A431 tumours to treatment with EGF receptor-blocking antibodies is associated with an elevated expression of VEGF (Viloria-Petit *et al*, 2001). Such cells, xenografted in nude mice, provide a model of highly VEGF-dependent (Melnyk *et al*, 1996) and aggressive tumour growth in an *in vivo* system. Since A431 cells have been recently described to express the VEGF_165_-binding sites (Li *et al*, 2001), we explored also the possible effect of CMDB7 on radiolabelled VEGF binding. We demonstrate that CMDB7 acts on both tumour and endothelial cells, decreasing in a potent manner the tumour growth and angiogenesis *in vivo*.

## MATERIALS AND METHODS

### Dextran derivative preparation

A water-soluble dextran derivative (CMDB7) was prepared as previously described (Chaubet *et al*, 1995). Its chemical composition, determined by acidimetric titration and elementary analysis of nitrogen, is 0% dextran, 70% carboxymethyl and 30% benzylamide. Average molecular weight was estimated as 80 000 g mol^−1^.

### Cell lines and cell culture

Human epidermoid carcinoma cell line (A431) and human umbilical vein endothelial cell line (HUV-EC-C) were purchased from the American Type Culture Collection (ATCC, Rockville, MD, USA). A431 cells were routinely grown in DMEM (Life Technologies, Inc., Gaithersburg, MD, USA) and HUV-EC-Cs in M199 (Life Technologies, Inc.) and were cultured at 37°C in a 5% CO_2_-humidified atmosphere. Both culture media were supplemented with 10% foetal calf serum (FCS), 2 mM L-glutamine, 1 mM sodium pyruvate, 50 U ml^−1^ penicillin and 50 *μ*g ml^−1^ streptomycin (all obtained from Life Technologies, Inc.). The cells were free of mycoplasma, bacteria and viruses.

### Preparation of conditioned media (CMs)

To assess the production of VEGF-A, the A431 cells at three different density were seeded into a 24-well culture plate (Falcon, Strasbourg, France) in DMEM supplemented with 10% FCS for 24 h. To obtain CM containing only the growth factors secreted by A431, the cells were washed twice with PBS, and incubated in 1 ml of serum-free DMEM containing 0.1% BSA (Sigma, St Louis, MO, USA). At the indicated time, the media were collected, cleared by centrifugation, and stored at −80°C before use. For other experiments, the cells were grown in 150 mm-diameter Petri dishes (Falcon) to 80% confluence in DMEM/10% FCS, washed and incubated in 10 ml dish^−1^ of serum-free medium.

### Determination of VEGF-A concentration in the A431-CMs by radioimmunoassay

The surface of flat-bottomed polystyrene wells (Disposable Immulon 1 Remowawell, Dynatech, Cambridge, MA, USA) were coated overnight at 4°C with 200 *μ*l of PBS buffer containing 50 ng polyclonal neutralising anti-VEGF IgG (R&D Systems, Abingdon, UK). The nonspecific interactions were saturated with PBS containing 0.1% BSA and 0.01% Tween-20 (PBT buffer) for an additional overnight at 4°C. After blocking, the wells were washed three times with 300 *μ*l of PBT buffer. Then, A431-CM or VEGF_165_ (R&D Systems) as standard at increasing concentrations (0–250 ng ml^−1^) and 50 pM ^125^I-VEGF_165_ (Amersham Pharmacia Biotech, Orsay, France) were added to a final volume of 200 *μ*l in PBT buffer. After an overnight incubation at 4°C, wells were washed three times with 300 *μ*l of PBT buffer and the radioactivity remaining in each well was measured in a *γ*-counter (LKB 1261 Multigamma).

### A431-CM effects on HUV-EC-C proliferation

HUV-EC-Cs were seeded at a density of 2 × 10^4^ well^−1^ into 24-well tissue culture plates (Falcon) in M199-10% FCS. After 24 h, the cells were growth arrested by serum starvation for another 24 h. Then, the cells were incubated for 48 h with A431-CM diluted in a serum-free medium to a final VEGF concentration of 10 ng ml^−1^ (concentration at which VEGF_165_ has the maximal mitogenic effect on HUV-EC-Cs (Hamma-Kourbali *et al*, 2001) in the presence or absence of 5 *μ*M CMDB7 (optimal concentration at which CMDB7 completely prevents the VEGF_165_ mitogenic effect on HUV-EC-Cs (Hamma-Kourbali *et al*, 2001) or 1 *μ*g ml^−1^ anti-human VEGF neutralising antibody (Sigma) characterised by neutralisation dose_50_=0.01–0.1 *μ*g ml^−1^. Cells were washed with PBS, dissociated with 0.025% trypsin-EDTA (Life Technologies) and counted using a Coulter counter (Coultronics, Margency, France). All experiments were performed in triplicate and data illustrate the mean cell numbers±s.e. provided from one representative of three independent experiments.

### A431 proliferation assay

A431 cells were seeded at a density of 10^4^ cells well^−1^ into 24-well tissue culture plates (Falcon) in DMEM–10% FCS and allowed to adhere to the plastic for 24 h. After washing with DMEM, the cells were incubated with CMDB7 at the indicated concentrations (day zero) in DMEM–1% FCS. At different times, cells were washed with PBS, dissociated with 0.025% trypsin-EDTA (Life Technologies, Inc.) and counted using a Coulter counter (Coultronics). In each case, samples were performed in triplicate, and data illustrate mean cell numbers±s.e. of one representative of three independently performed experiments.

### VEGF_165_ binding to A431 cells

For displacement binding assays, A431 cells were grown until confluence on 24-well tissue culture plates (Falcon). After an overnight incubation in serum-free medium and two washings with ice-cold binding buffer (PBS/0.1% BSA), the cells were incubated with 7 pM ^125^I-VEGF_165_ (Amersham, Pharmacia Biotech) and CMDB7 at various concentrations from 8 × 10^−9^ to 4 × 10^5^ M at 4°C for 2 h. Incubation was terminated by gently aspirating the medium and washing the cell monolayer three times with ice-cold binding buffer. After cell solubilisation in 0.3 ml of 0.5 N NaOH, the bound radioactivity was measured in a *γ*-counter (LKB 1261 Multigamma). Nonspecific binding was determined in the presence of an excess (5000 pM) of unlabelled VEGF_165_ (R&D Systems).

### Tumour cell inoculation in nude mice

All *in vivo* experiments were carried out with ethical committee approval and met the standards required by the UKCCCR guidelines (Workman *et al*, 1998). A431 cells (5 × 10^6^) were inoculated s.c. in the right flank of 4-week-old athymic nude mice (nu/nu) (Charles River Laboratory, Aubin-les-Elbeuf, France). The animals (*n*=20) were kept in a temperature-controlled room on a 12 h : 12 h light–dark schedule with food and water *ad libitum*. All mice developed single s.c. palpable tumours of approximately 50 mm^3^ 6 days after cell inoculation. Then, mice were placed in control (*n*=10) and CMDB7-treated groups (*n*=10). Mice were treated by subcutaneous (s.c.) injection of 0.1 ml PBS alone (control) or containing 10 mg kg^−1^ CMDB7 close to the tumour, three times a week for 2 weeks. Tumours were measured along two major axes with a calliper. Tumour volume was calculated as follows:

*V* = (4/3)*πR*_1_^2^*R*_2_

where *R*_1_ is radius 1, *R*_2_ is radius 2, and *R*_1_ <*R*_2_.

### Tissue preparation and immunohistochemical staining

Immediately after surgical resection, the tumour specimens were fixed with 4% paraformaldehyde and processed to paraffin inclusion. The intratumour mice endothelial cells were specifically stained with GSL-1 lectin (Vector Laboratories, Burlingame, CA, USA) in 5-*μ*m sections as previously described (Bagheri-Yarmand *et al*, 1999). The GSL-1 lectin binds specifically to galactosyl residues present on vascular endothelium in mice (Alroy *et al*, 1987; Mattsson *et al*, 2002). The proliferative index of tumour xenograft was determined by human Ki-67 staining with monoclonal mouse antibody (MIB-1; 1 : 50; Dako, Trappes, France). The epitope retrieval was performed in 10 mM citrate buffer pH=6.0 for 40 min at 98°C. Specific reactions were visualised with 3,3′-diaminobenzidine (DAB) as chromogen.

### Image analysis

For each GSL-1- or MIB-1-labelled section of control or CMDB7-treated tumour, five fields containing exclusively viable tumour cells, as indicated by the haematoxylin staining, were selected randomly for analysis. Image analysis was performed using the NIH programme (developed at NIH and available on the Internet at http://rsb.info.nih.gov/nih-image/). The endothelial cell density in each field was expressed as the ratio of endothelial cell area and the total viewed area × 100 (%). To determine the proliferative index, we estimated the percentage of tumour cell nuclei positive for Ki-67 marker. These values were then averaged for untreated (control) and treated-CMDB7 tumours.

### Statistical analysis

Multiple statistical comparisons were performed using ANOVA in a multivariate linear model. Statistical comparisons were conducted using the Mann–Whitney *t*-test. *P*<0.05 was considered statistically significant.

## RESULTS

### CMDB7 inhibits, like neutralising anti-VEGF_165_ antibody, mitogenic effect of A431-CM on HUV-EC-Cs

According to previous studies (Melnyk *et al*, 1996), we found that A431 cells secrete in the culture medium large amounts of VEGF-A. Moreover, we showed here that VEGF production is cell number- and time-dependent (Table 1

**Table 1:** Concentration of VEGF in the conditioned media of A431 tumour cells

**Cell number**	**24 h**	**48 h**
2.5 × 10^4^	<0.4	1
5 × 10^4^	2	4
10^5^	8	16

A431 cells were seeded at the indicated density into 24-well plate in DMEM–10% FCS medium for 24 h. After washing, they were incubated in serum-free medium. At the indicated time, the media were collected. VEGF-A concentration was asessed by radio immunoassay as described in Materials and Methods. Values are in ng ml^−1^. The limit of sensitivity of the assay was 0.4 ng ml^−1^.

).

**Figure 1:**
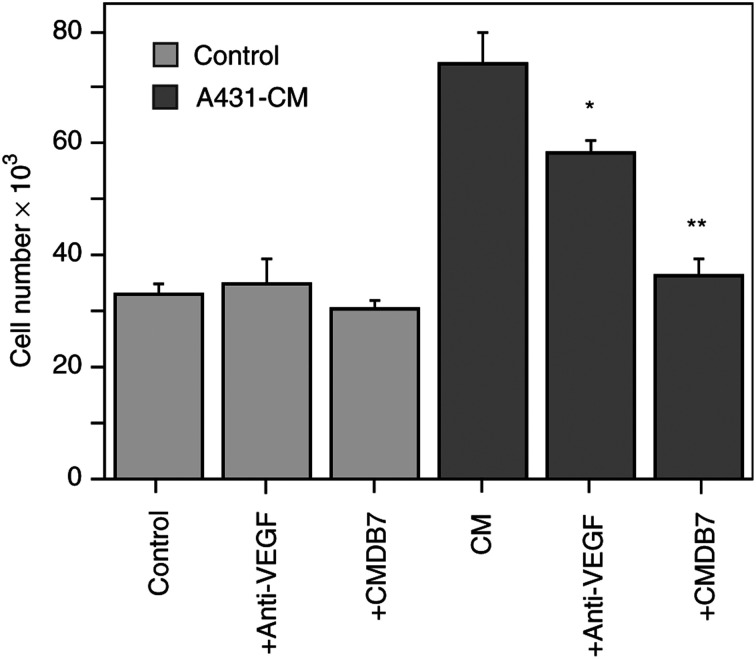
CMDB7 inhibits A431-CM mitogenic effect. Quiescent HUV-EC cells were incubated with A431-CM with or without 5 *μ*M CMDB7 or 1 *μ*g ml^−1^ anti-human VEGF neutralising antibody. After 48 h, the cells were trypsinised and counted using a Coulter counter. The values represent mean cell numbers±s.e. (bars), obtained in triplicate in one of the three independent experiments.

As expected, A431-CM stimulated the *in vitro* proliferation of HUV-EC-Cs by 2.5-fold after 48 h of incubation (Figure 1

). This mitogenic effect is, at least in part, VEGF-specific since the neutralising antibodies against recombinant VEGF inhibited the A431-CM-induced proliferation of HUV-EC-Cs by 45% after 48 h treatment. A431-CM, used in this experiment, contained 10 ng ml^−1^ of VEGF_165_ as revealed by specific radioimmunoassay. At the same concentration, recombinant VEGF_165_ has a similar mitogenic effect on HUV-EC-Cs (Hamma-Kourbali *et al*, 2001), as described above the addition of 5 *μ*M CMDB7 prevented the stimulatory effect of A431-CM on HUV-EC proliferation (Figure 1). When HUV-EC-Cs were cultivated in serum-free medium, CMDB7 or neutralising anti-VEGF_165_ antibodies had no effect.

### CMDB7 inhibits A431 cell proliferation *in vitro*

**Figure 2:**
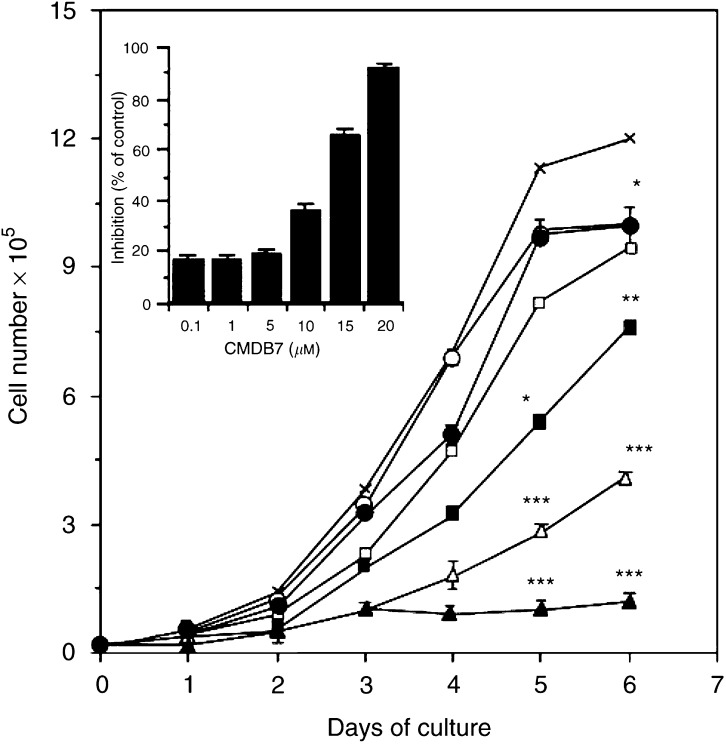
Inhibition of A431 cell growth *in vitro*. A431 cells were seeded at 10^4^ cells well^−1^ in 24-well plates in DMEM containing 10% FCS. On the following day (day 0), the medium was changed to DMEM containing 1% serum (**x**) and 0.1 *μ*M (○), 1 *μ*M (•), 5 *μ*M (□), 10 *μ*M (▪), 15 *μ*M (▵), or 20 *μ*M (▴) CMDB7. At the indicated time, cells were trypsinised and counted. The inset shows the percentages of inhibition of the A431 cell growth by CMDB7 at increasing concentrations at day 6. The values represent mean cell numbers±s.e. (bars), obtained in triplicate in one of the three independent experiments.

Next, we tested CMDB7 for its ability to affect the *in vitro* growth of A431 tumour cells. We demonstrated that treatment with CMDB7 at increasing concentrations, ranging from 0.1 to 20 *μ*M, resulted in a concentration- and time-dependent inhibition of A431 cell number (Figure 2

).

In contrast, 1 *μ*g ml^−1^ anti-VEGF antibody had no effect on A431 proliferation *in vitro* (data not shown) as reported by others (Melnyk *et al*, 1996).

### CMDB7 inhibits VEGF_165_ binding to A431 tumour cells

**Figure 3:**
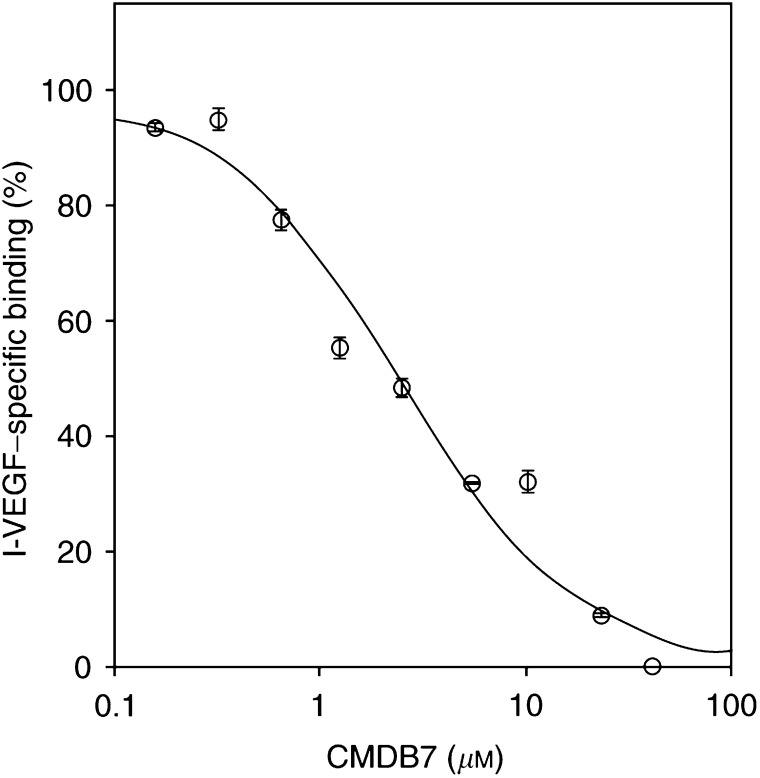
CMDB7 inhibits VEGF_165_ binding to A431 cells. Confluent A431 cells were incubated for 2 h at 4°C in the presence of 7 pM ^125^I-VEGF_165_ and CMDB7 at the indicated concentrations (logarithmic scale). Nonspecific binding was determined in the presence of 5000 pM unlabelled VEGF_165_. Results are expressed as the mean±s.e. (bars) of experiments done in duplicates and repeated at least twice.

Since A431 cells produce VEGF-A and binds VEGF_165_ on the surface (Li *et al*, 2001), we explored if CMDB7 is able to compete for VEGF_165_-specific binding (Figure 3

). CMDB7 decreased the ^125^I-VEGF_165_-specific binding to A431 cells at concentrations ranging from 0.1 to 50 *μ*M with a half-maximum inhibitory effect (IC_50_) at concentration 2 *μ*M.

### CMDB7 inhibits the growth of A431 cells xenografted in nude mice

**Figure 4:**
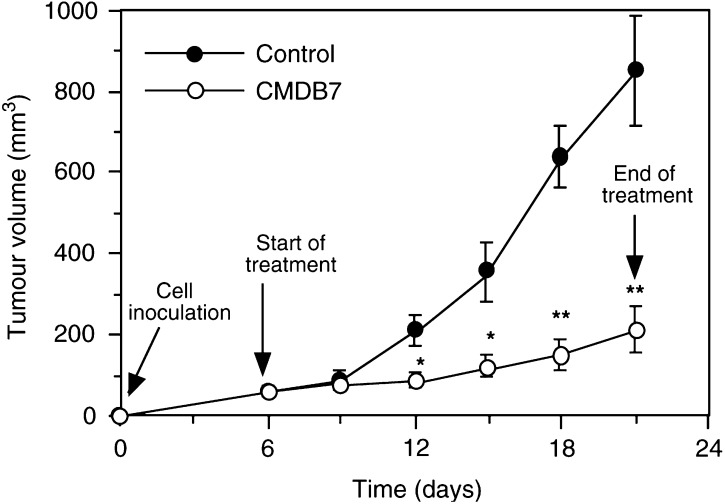
CMDB7 inhibits primary tumour growth. A431 carcinoma cells (5 × 10^6^) were inoculated s.c. into the right flank of female nude mice. When tumour volume reached 100 mm^3^ (6 day), CMDB7 (10 mg kg^−1^) was administrated s.c. three times a week for 2 weeks. Tumours were measured and the results are presented as the mean tumour volume ±s.e. (bars) obtained from 10 mice in each group, *P*<0.001; CMDB7-treated group *vs* controls.

The tumours appeared in 100% of mice 6 days after A431 cell inoculation. CMDB7 inhibited the growth of A431 tumours by 73% (*P*<0.001) after 2 weeks of treatment (Figure 4

).

No apparent toxicity was noticed during treatment with CMDB7. No signs of toxicity such as diarrhoea, infection, weakness or lethargy were observed. The body weight of the inoculated mice was not affected by CMDB7 after 2 weeks of treatment. All treated mice were alive at the end of treatment.

### CMDB7 decreases the proliferative index of A431 xenografts

The specific Ki-67 staining was less intense in CMDB7-treated tumours as compared to control (nontreated) ones. The proliferative index for treated and control xenografts were significantly (*P*=0.05) diffferent, 26±8 and 34±10%, respectively (mean±s.e.m). These data suggest that CMDB7 inhibited directly *in vivo* the proliferation of tumour cells. In all xenografts, treated as well as nontreated, the areas of necrosis/apoptosis were large, but localised in the centre of tumour. There did not appear to be obvious differences in the degree of necrosis observed in both cases. We had no difficulties in obtaining five fields of viable cells in all tumours.

### CMDB7 inhibits the intratumour endothelial cell density

**Figure 5:**
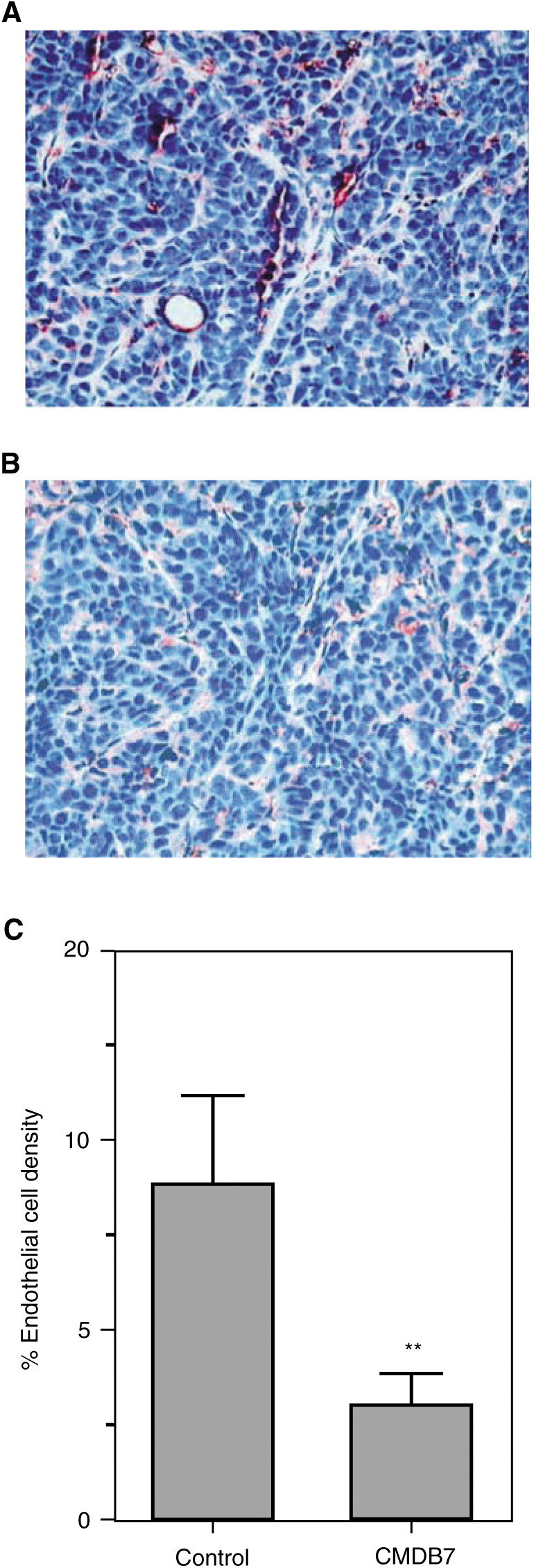
CMDB7 inhibits the tumour angiogenesis. Intratumour vascularisation was analysed immunohistologically after labelling of endothelial cells with a specific marker GSL-1. (**A**) Section of saline-treated (control) tumour and (**B**) section of tumour treated with 30 mg kg week^−1^ of CMDB7. (**C**) Endothelial cell density was evaluated by image analysis of GSL-1-labelled endothelial cells and the results are presented as the mean areas±s.e. (bars) of endothelial cells in the CMDB7-treated and control tumour sections obtained from 10 mice for each group. ^**^, significantly different from control (*P*<0.001).

Selective GSL-1 staining showed that CMDB7 treatment reduced the endothelial cell quantity in tumour tissue (Figure 5B

) as compared to control (Figure 5A). The mean percentage of endothelial cell area (endothelial cell density) in viable fields of CMDB7-treated tumours (2.9 ± 0.6; 50 fields in 10 tumours) was inhibited by 66% (*P*<0.001) as compared to control tumour value (8.6±0.7; 50 fields in 10 tumours) (Figure 5C).

## DISCUSSION

Antiangiogenesis is a promising therapeutic approach for the treatment of cancer (Folkman, 1995; Schweigerer, 1995). VEGF plays a crucial role in tumour angiogenesis and the inhibition of VEGF action decreases tumour growth *in vivo* (Kim *et al*, 1993; Goldman *et al*, 1998; Lin *et al*, 1998). Since the human A431 carcinoma cells secrete high amounts of VEGF (Myoken *et al*, 1991) and develop in nude mice tumours whose growth is highly VEGF-dependent (Melnyk *et al*, 1996), they provide a good model to test the availability of molecules that inhibit VEGF bioactivity. In this study, we assessed the anti-VEGF activity of CMDB7, described recently *in vitro* (Hamma-Kourbali *et al*, 2001), on A431 xenografted in nude mice, an extremely aggressive tumour model. CMDB7 is, to our knowledge, the only one of heparin analogues reported to be efficient in A431 xenograft model.

This study demonstrated that the s.c. injection of viable A431 cells yielded a 100% tumour uptake rate. The 2 week treatment with CMDB7 resulted in a 73% tumour growth inhibition associated with a 66% decrease in endothelial cell density. Compared to the human breast MDA-MB-435 (Bagheri-Yarmand *et al*, 1999) and MCF-7ras (Bagheri-Yarmand *et al*, 1998b) tumours, the magnitude of response of the A431 tumours to CMDB7 treatment was greater. Here, we observed that rapid A431 tumour growth was associated with high intratumour endothelial cell density, suggesting a direct relation between vascularisation within the primary tumour and the tumour growth rate. In 3-week-old A431 control (untreated) tumours, the endothelial cell density was 8.6%, while in 12-week-old MDA-MB-435 and MCF-7ras xenografts this value was 4.9% (Bagheri-Yarmand *et al*, 1999) and 6.1% (Bagheri-Yarmand *et al*, 1998b), respectively. Our observations are in agreement with results of Kim (1993), which demonstrated that the inhibitory effect of anti-VEGF antibody on tumour growth was more pronounced in the case of human A673 rhabdomyosarcoma secreting the highest quantity of VEGF and giving the most rapidly growing tumours as compared to G55 glioblastoma and SK-LMS-myosarcoma. In the CMDB7-treated tumours, a reduction of 66% in the density of endothelial cells indicates that this treatment attenuated the rate of neovascularisation, but did not completely reverse the initial activation of angiogenesis. The augmentation of CMDB7 dose did not result in increased efficiency of the drug *in vivo* (data not shown). Our results demonstrate that CMDB7 inhibited A431 tumour growth by, at least in part, decreasing intratumour endothelial cell density. The mechanism of CMDB7 action on endothelial cells is probably not direct and involves, as we recently described *in vitro* (Hamma-kourbali *et al*, 2001), a direct interaction of the drug with VEGF_165_ that becomes unavailable for specific receptors. In agreement, we demonstrate here that CMDB7 inhibits the A431-CM stimulation of endothelial cell proliferation.

The other mechanism by which CMDB7 reduced the A431 tumour growth is direct inhibition of A431 cell proliferation as evidenced by a decrease of proliferative index in treated xenografts compared to nontreated ones. In this study, we demonstrated that CMDB7 inhibited, like VEGF, the binding of ^125^I-VEGF_165_ to A431 cells with IC_50_ similar to the concentration at which CMDB7 inhibits efficiently the A431 proliferation *in vitro*. These findings could argue for the possible autocrine mitogenic action of VEGF on A431 cells. However, the depletion of VEGF amount in A431-conditioned medium by anti-VEGF antibody did not affect the A431 proliferation, although it did inhibit endothelial cell growth. It suggests that VEGF binding sites on the A431 cell surface are not involved in classical, KDR-dependent transmission of mitogenic signal. The A431 growth decrease by CMDB7 *in vitro* could involve the inhibition of other mitogenic growth factors. This interpretation can be strengthened by our previous studies demonstrating that CMDB7 inhibited the activity of heparin-binding PDGF and TGF*β* by altering their conformation, but did not change the activity of EGF and IGF1, which are not heparin-binding growth factors (Bagheri-Yarmand *et al*, 1997,1998a,1998b). Independently, the possible VEGF autocrine pathway in A431 could mediate tumour cell survival by protecting them from apoptosis as it was recently reported for breast cancer MDA-MB-231 cells (Bachelder *et al*, 2001). Further studies are necessary to understand the mechanisms of direct CMDB7 inhibitory action on A431 proliferation *in vitro*.

Altogether, our findings demonstrate that CMDB7 has a strong antiangiogenic and antitumour action *in vivo*, also when tumour cells produce a high level VEGF and EGFRs. CMDB7 acts directly on both tumour and endothelial cells, decreasing in a potent manner the tumour growth by increasing the proliferation of tumour cells and especially angiogenesis *in vivo*. The development of resistance to antiangiogenic drugs is becoming apparent (Kerbel *et al*, 2001). It is very important, now, to enlarge the diversity of molecular targets for antiangiogenic drugs and to use a combination of antiangiogenic therapies. One of the possible mechanisms of this resistance may be due to redundancy of different proangiogenic growth factors made by tumour cells. When one angiogenic factor is targeted, the cancer cells increase production of other angiogenic factors. In this context, we believe that the ability of CMDB7 to interact with several angiogenic factors, including VEGF (Hamma-Kourbali *et al*, 2001), bFGF (Bagheri-Yarmand *et al*, 1997,1998a), TGF-*β* and PDGF (Bagheri-Yarmand *et al*, 1998b), will permit to oppose or at least put off the development of resistance. Recently, it was reported that the resistance of tumours to treatment with EGF receptor-blocking antibodies can be associated with an elevated expression of VEGF (Viloria-Petit *et al*, 2001). Since we show in this study that CMDB-7 efficiently blocks *in vivo* the effects of VEGF produced at high level, we can speculate that this drug could be useful in the case of failure to anti-EGFR treatment. It is believed now that because angiogenesis is a complex and multistage process, treatment with more than one antiangiogenic agent may be beneficial (Cherrington *et al*, 2000). Also, the neutralisation of angiogenic growth factors, especially VEGF, in tumour with CMDB7 may increase the effects of a variety of antiangiogenic inhibitors (Kerbel *et al*, 2001). For example, the reduced ability of Taxotere to induce apoptosis of endothelial cells in the presence of VEGF (Sweeney *et al*, 2001) could be restored by combined treatment with CMDB7. CMDB7 can be used not only as monotherapy but also especially in combination with other antiangiogenic and anticancer drugs to cause acute tumour regression by delaying development of resistance and by enhancing the effects of other drugs.
